# Morphological and molecular identification of arrhenotokous strain of *Diglyphuswani* (Hymenoptera, Eulophidae) found in China as a control agent against agromyzid leafminers

**DOI:** 10.3897/zookeys.1071.72433

**Published:** 2021-11-17

**Authors:** Su-Jie Du, Zoya Yefremova, Fu-Yu Ye, Chao-Dong Zhu, Jian-Yang Guo, Wan-Xue Liu

**Affiliations:** 1 State Key Laboratory for Biology of Plant Diseases and Insect Pests, Institute of Plant Protection, Chinese Academy of Agricultural Sciences, Beijing, 100193, China Institute of Plant Protection, Chinese Academy of Agricultural Sciences Beijing China; 2 Steinhardt Museum of Natural History, Department of Zoology, Tel Aviv University, Ramat Aviv, 69978, Israel TelAviv University Ramat Aviv Israel; 3 State Key Laboratory of Zoological Systematics and Evolution, Institute of Zoology, Chinese Academy of Sciences, Beijing, 100101, China Institute of Zoology, Chinese Academy of Science Beijing China

**Keywords:** Arrhenotoky, Diglyphuswani, morphology, phylogeny, thelytoky

## Abstract

*Diglyphus* species are ecologically and economically important on agromyzid leafminers. In 2018, a thelytokous species, *Diglyphuswani* Liu, Zhu & Yefremova, was firstly reported and described. Subsequently, the arrhenotokous *D.wani* were discovered in Yunnan and Guizhou Provinces of China. We compared the morphological characteristics of thelytokous and arrhenotokous strains. However, the females of two strains had a strongly similar morphology and showed subtle differences in fore- and hind-wings. The difference was that forewing of arrhenotokous female was with denser setae overall, showing that costal cell with 2 ~ 4 rows of setae on dorsal surface and the setae of basal cell with 15 ~ 21 hairs and forewing of thelytokous female was with two rows of setae on dorsal surface and basal cell with 10 ~ 15 hairs generally. The setation beneath the marginal vein of the hind-wing of arrhenotokous female is denser than the same area of thelytokous female. To explore the genetic divergence between thelytokous and arrhenotokous strains of *D.wani*, the mitochondrial and nuclear gene were applied and sequenced. The polygenic analyses revealed that two strains can be distinguished by COI, ITS1 and ITS2. The mean sequence divergence between the two strains was 0.052, 0.010 and 0.007, respectively. Nevertheless, the 28S gene was unfeasible due to its containing a sharing haplotype between different strains. The two strains of *D.wani* are dominant parasitoids against agromyzid leafminers and such effective discernible foundation provides future in-depth studies on biological characteristics, along with insight into field application of two strains of *D.wani*.

## Introduction

Agromyzidae belongs to Diptera and is a family consisting of about 2750 species (Tschirnhaus et al. 2000) and approximately 110 species of them are known to be the main pests of cultivated crops world-wide (Dempewolf 2020). In China, over 130 Agromyzidae species have been reported. Of these, at least six species, including indigenous *Chromatomyiahorticola*, *Liriomyzachinensis* and invasive *L.sativae*, *L.huidobrensis*, *L.trifolii* and *L.bryoniae*, are major agricultural leaf-mining pests, especially on vegetables (Kang et al. 2009; Liu et al. 2013). For decades, the main prevention for agromyzid leafminers has been chemical control with pesticides (Kang et al. 2009). With the frequent use and abuse of chemical pesticides, agromyzid leafminers have gradually developed resistance to insecticides (Parrella and Keil 1984; Tokumaru and Yamashita 2004) and natural enemies have decreased (Trumble and Toscano 1983; Hernández et al. 2011). Therefore, it requires sustainable, effective and biocontrol strategies to regulate the damage of agromyzid leafminers. Notably, applying Hymenoptera parasitoids are considered to be primary strategies, because these species are the most effective natural enemies against agromyzid leafminers (Parrella 1987; Liu et al. 2009; Mujica and Kroschel 2011; Ridland et al. 2020).

*Diglyphus* (Hymenoptera: Eulophidae) are economically-important parasitoids against agromyzid leafminers (Zhu et al. 2000; Yefremova et al. 2011; Liu et al. 2013; Hansson and Navone 2017), although there are a few species (e.g. *D.begini*, *D.chabrias*, *D.isaea*) that attack other hosts, such as Lepidoptera, Lyonetiidae and Nepticulidae (Noyes 2019). Hitherto, 40 species placed within genus *Diglyphus* have been reported all over the world (Zhu et al. 2000; Hansson and Navone 2017; Ye et al. 2018) and 17 species are distributed in China (Zhu et al. 2000; Liu et al. 2013; Ye et al. 2018). Several *Diglyphus* species (e.g. *D.isaea* and *D.begini*) exhibited strong biological control capability and were released to regulate the population of agromyzid leafminers (Boot et al. 1992; Heinz et al. 1993).

In Hymenoptera parasitoids, some species have two reproduction modes: (1) arrhenotoky, where haploid males arise from unfertilised eggs and diploid females from fertilised eggs and (2) thelytoky, which is obligate parthenogenesis and produces only female progenies or occasional males (Heimpel and de Boer 2008). Amongst *Diglyphus* species, a thelytokous parasitoid named *D.wani* was firstly reported and displayed favourable biocontrol potential showing three types of host-killing behaviour (host-feeding, parasitism and host-stinging) (Ye et al. 2018).

In arthropods with haplodiploid sex determination mechanism, thelytokous strains may exist with their corresponding arrhenotokous strains (van der Kooi et al. 2017). In Eulophidae, several species with two strains (reproduction modes) have been reported, such as *Neochrysocharisformosa* (Adachi-Hagimori et al. 2011; Yang et al. 2017) and *Pnigaliosoemius* (Gebiola et al. 2012). For *D.wani*, whether there is also an arrhenotokous strain is not clear. In the field investigations, we firstly discovered arrhenotokous *D.wani* in Yunnan Province of China, which was a dominant parasitoid on agromyzid leafminers and established a stable colony in the laboratory. We preliminarily attempted to make a morphological distinction, but two strains of *D.wani* were likely to be so similar that it would be difficult to discriminate each other accurately. However, accurate identification was essential for potential application of *D.wani*. Thus, in addition to traditional morphological classification, molecular methods were also adopted, because multiple gene markers, such as the cytochrome *c* oxidase subunit I gene (COI) and nuclear internal transcribed spacers (ITS1 and ITS2), have been also applied widely for species identification (Campbell et al. 1993; Chen et al. 2004; Sha et al. 2006; Munro et al. 2011; Om et al. 2017; Ye et al. 2018).

In this paper, the combination of morphological and molecular tools (COI, ITS1, ITS2 and 28S) was applied to characterise and compare differences between arrhenotokous and thelytokous strains of *D.wani*. The results will promote the future biocontrol application of two strains of *D.wani*.

## Materials and methods

### Sampling

Sampling of the parasitoids on agromyzid leafminers was conducted in the different geographical regions of China as described in Table 1. The collected individuals of *D.wani* were 40 thelytokous individuals (Qinghai: 15♀; Hebei: 16♀; Tibet: 9♀) and 54 arrhenotokous individuals (Yunnan: 20♀+9♂; Guizhou: 19♀+6♂). *D.isaea* (Beijing: 3♀) and *D.crassinervis* (Jilin: 5♀) were also collected for phylogenetic data (Table 1). The collected samples were carefully labelled and kept individually according to the different locations. All specimens from plant leaves infested with parasitised leafminer larvae were maintained in climate chambers set to 25 ± 1°C, relative humidity of 30 w~ 50% and a photoperiod of 14 h: 10 h (light: dark) until parasitoids emerged.

**Table 1.** Specimens collected from leaves damaged by *Chromatomyiahorticola* in China, 2018.

Species	Sex	Plants	Locality	Coordinates
Arrhenotokous *D.wani*	5♀+ 2♂	*Pisumsativum*	Guiyang, Guizhou	26°37'N, 106°36'E
	9♀+ 4♂	*Pisumsativum*	Guiyang, Guizhou	26°34'N, 106°43'E
	5♀	*Brassicanapus*	Guiyang, Guizhou	26°34'N, 106°43'E
	8♀+ 3♂	*Brassicanapus*	Kunming, Yunnan	24°53'N, 102°47'E
	8♀+ 6♂	*Brassicanapus*	Kunming, Yunnan	25°00'N, 102°45'E
	4♀	*Gypsophilapaniculata*	Kunming, Yunnan	25°00'N, 102°45'E
Thelytokous *D.wani*	9♀	*Pisumsativum*	Lhasa, Tibet	29°38'N, 91°02'E
	8♀	*Raphanussativus*	Xining, Qinghai	36°39'N, 101°36'E
	2♀	*Brassicanapus*	Xining, Qinghai	36°39'N, 101°36'E
	5♀	*Brassicanapus*	Xining, Qinghai	36°43'N, 102°45'E
	6♀	*Orychophragmusviolaceus*	Zhangjiakou, Hebei	40°46'N, 114°52'E
	5♀	*Pisumsativum*	Zhangjiakou, Hebei	40°46'N, 114°52'E
	5♀	*Pisumsativum*	Zhangjiakou, Hebei	40°58'N, 115°17'E
*D.isaea*	3♀	*Pisumsativum*	Beijing	39°56'N, 116°20' E
*D.crassinervis*	5♀	*Alliumfistulosum*	Gongzhuling, Jilin	43°50'N, 124°82'E

### Morphological Identification

The collected parasitoid samples were transferred to plastic tubes filled with 99.7% ethanol and then stored at -20°C for subsequent classification. These samples were examined with a stereomicroscope (Olympus Corporation, SZX-16, Tokyo, Japan). Terminology and measurement methods referred to Gibson (2003). The abbreviations used are: F1-F2, first to second flagellomeres; SMV, MV, PMV and STV, which are submarginal, marginal, post-marginal and stigmal veins; OOL, the minimum distance between an eye margin and the adjacent posterior ocellus; and POL, the minimum distance between the posterior ocelli. Measurements of body, gaster and ovipositor lengths were taken using an optical microscope (Keyence Corporation, VHX-2000, Tokyo, Japan). Relative measurements were used for the other parts. The ratio of gaster to ovipositor was calculated in Microsoft Excel 2016 using Mean ± SD (standard deviation). Photographs of arrhenotokous and thelytokous *D.wani* were taken by an Olympus CX31 microscope and an Olympus BX43 microscope with a Helicon Focus system, respectively. Of *Diglyphus* parasitoids that we surveyed, *D.crassinervis* was close to *D.wani* relatively in terms of morphology. Additionally, *D.isaea* was a common parasitoid on agromyzid leafminers. We selected the two species to discover further phylogenetic relationships between them and *D.wani*.

### Molecular diagnosis

#### Parasitoid DNA extraction

Using the QIAGEN blood or tissue genome kit (Germany) we followed the steps according to the manufacturer’s standard protocol of kit to extract DNA of a single parasitoid. The DNA was stored at -20°C for molecular research.

#### Amplification and sequencing of gene fragments

This study used primers COISF (5'-TAAGATTTTGATTATT(AG)CC(TA)CC-3') (Sha et al. 2006) and COI2613 (5'-ATTGCAAATACTGCACCTAT-3') (Chen et al. 2004) to amplify the parasitoid COI gene fragment. ITS1 primers were 18sf1 (5’-TACACACCGCCCGTCGCTACTA-3’) and 5p8sB1d (5’-ATGTGCGTTCRAAATGTCGATGTTCA-3’) (Ji et al. 2003). Primers ITS2F (5’-TGTGAACTGCAGGACACATG-3’) and ITS2R (5’-AATGCTTAAATTTAGGGGGTA-3’) (Campbell et al. 1993) were used to amplify the parasitoid ITS2 gene fragment. Primers D2F (5’-AGTCGTGTTGCTTGATAGTGCAG-3’) and D2R (5’-TTGGTCCGTGTTTCAAGACGGG-3’) (Campbell et al. 1993) were used to amplify the D2 region of the 28S gene fragment of parasitoids.

The PCR reaction systems were that, 0.4 μl *Taq* enzyme (2.5 Uμl^-1^), 0.4 μl dNTP (2.5 mM), 2.5 μl 10× buffer (containing Mg^2+^), 0.4 μl forward primer, 0.4 μl reverse primer, 50 ng DNA template and adding ddH_2_O to 25 μl finally. The primer annealing temperatures of COI, ITS1, ITS2 and 28S were 48°C, 58°C, 52°C and 58°C, respectively. The rest of the programmes were set uniformly and they were initial denaturation at 95°C for 3 min followed by 35 cycles of denaturation at 95°C for 15 s, annealing for 15 s, extension at 72°C for 60 s and a single cycle of final extension at 72°C for 5 min. The PCR instrument was an ABI thermal cycler (Veriti Applied Biosystems 9902, Singapore). At the same time, a negative control made sure the PCR amplification system was not contaminated.

After the PCR reaction, taking 4 µl of the PCR product, mixing it with 0.3 µl of 10× Loading buffer, then electrophoresing products in 1% agarose solution containing Gold View II (Solarbio, Beijing, China), setting voltage 100 V, current 400 mA and 30 minutes. After the electrophoresis, we observed the results in the gel imaging system and saved the photos. The PCR unpurified products containing the target bands were sent to Tsing Ke Biological Technology, Beijing of China, for Bi-directional sequencing.

When the gene sequence peak map showed double peaks in Bi-direction, the sequences needed to be cloned. After the PCR products were purified, the target fragments were ligated into the pEASY-T3 cloning vector (Transgen Biotech, Beijing, China) and transferred into *E.coli* competent cells Trans-T1 (Transgen Biotech, Beijing, China) according to the manufacturer’s instructions. Finally, using the universal M13 vector primer to detect whether the target fragments were successfully connected, each sample tested five positive clones to evaluate the difference between clones. In this study, the sequence divergence of clones of every sample was small about 0 ~ 0.003, usually about 0.001. Thus, we randomly selected a sequence for phylogenetic analysis.

#### Sequence analysis

All sequences were analysed by BLAST (Basic Local Alignment Search Tool) in the NCBI database to determine whether the amplified sequences belonged to mitochondria and nuclear genes. The sequences were aligned by using the CLUSTAL W tool of MEGA 7.0 (Kumar et al. 2016) and using the default options. Pairwise and mean sequence divergence, variation sites and parsimony informative sites were estimated, based on the Kimura-2 parameter (K2-P) (Kimura 1980). For COI, the sequences were translated into the amino acid sequence, based on the invertebrate mitochondrial genetic code so as to examine no stop codes. Then, version 5 of the DNASP(Librado and Rozas 2009)was used to calculate gene haplotypes.

#### Phylogenetic analysis

The phylogenic tree was constructed with UPGMA (the unweighted pair group method, based on arithmetic averages) methods, based on the K2-P model and were performed with MEGA 7.0 (Kumar et al. 2016). Bootstrap values were obtained after conducting 1000 replications for sequence divergence and phylogenetic relationships. Bootstrap support > 70% and taxonomically relevant splits, were indicated above branches of the phylogenic tree.

## Results

### Morphological description

#### Diglyphus wani

Taxon classificationAnimaliaHymenopteraEulophidae

Liu, Zhu & Yefremova, 2018

D9FA4C9B-B8E9-5EBA-B84B-183049E8F8F5

##### Type material

. The type specimens of arrhenotokous *D.wani* were deposited in the Institute of Plant Protection, Chinese Academy of Agricultural Sciences, Beijing, China.

##### Arrhenotokous male

**Figure 1.**
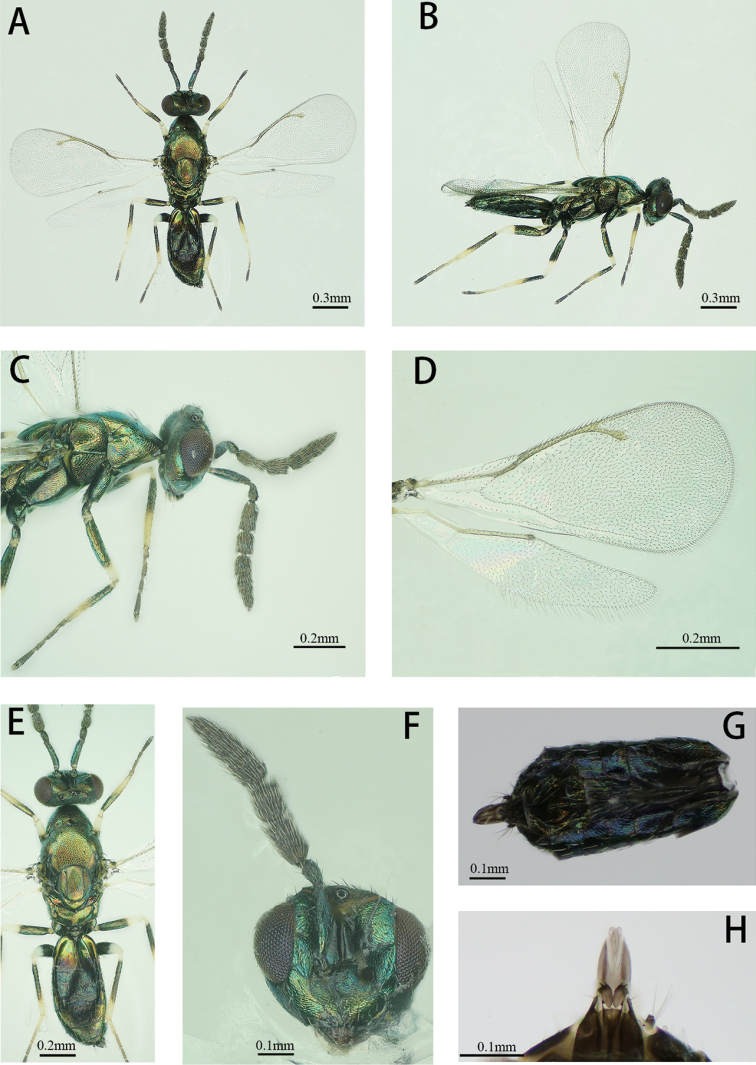
*Diglyphuswani*, arrhenotokous male **A** Body, dorsal view **B** Body, lateral view **C** Head and mesosoma, lateral view **D** Right fore and hind wing **E** Head, tergum and gaster, dorsal view **F** Head, front view **G** Metasoma, ventral view **H** Genitalia, ventral view.

(Figs 1A, B). Body length 1.0–1.9 mm, forewing length 0.9–1.2 mm. Body light green with a metallic tint; tegulae dark brown, antenna and mandibles brownish, labial and maxillar palpae pale yellow, compound eyes dark red. Legs with dark green and metallic coxae, brownish and metallic trochanters, anterior 3/4 to the middle of all femora dark brown and metallic, posterior pale yellow, all tibiae dark brown with metallic shine, except base and apical 1/5–2/5 part white or pale yellow, hind tibia with anterior surface dark to white-yellow and posterior surface dark, tarsi yellow, except last 4^th^ tarsomere (dark brown) and 3^rd^ tarsomere (brownish), wings hyaline.

**Antenna** (Fig. 1C). Antenna with scape 3.8× as long as broad, pedicel 2.1× as long as broad, 2 anelli, F1 1.9× as long as broad, F2 1.7× as long as broad, clava 3-segmented 3.4× as long as broad. F1 1.2× as long as F2, clava 1.7× as long as scape and 2.6× as long as F2.

**Head** (Figs 1C, F). Head wider than height. Toruli inserted a little above the level with the lower margin of eyes. Malar sulcus present, straight, mouth width 1.6× of malar space.

**Thorax** (Figs 1C, E). Pronotum, mesonotum and scutellum metallic green. Mesoscutum as long as scutellum. Scutellum 1.09 × as long as broad. Propodeum 2.8× as broad as long, smooth, without median carina.

**Wing** (Fig. 1D). Forewings 2.2× as long as broad. SMV tapering to apex, with six setae dorsally. Costal cell with three rows of setae, ~ 10 dorsal setae on anterior margin apically. Speculum is very small with sparse setations. Relative measurements: SMV: MV: PMV: STV = 10.6: 14.7: 4.7: 4.1.

**Metasoma** (Figs. 1G and 1H). Petiole short. Gaster 1.8–1.9× as long as broad. Genitalia: digitus with two developed and two reduced spines.

##### Arrhenotokous female.

**Figure 2.**
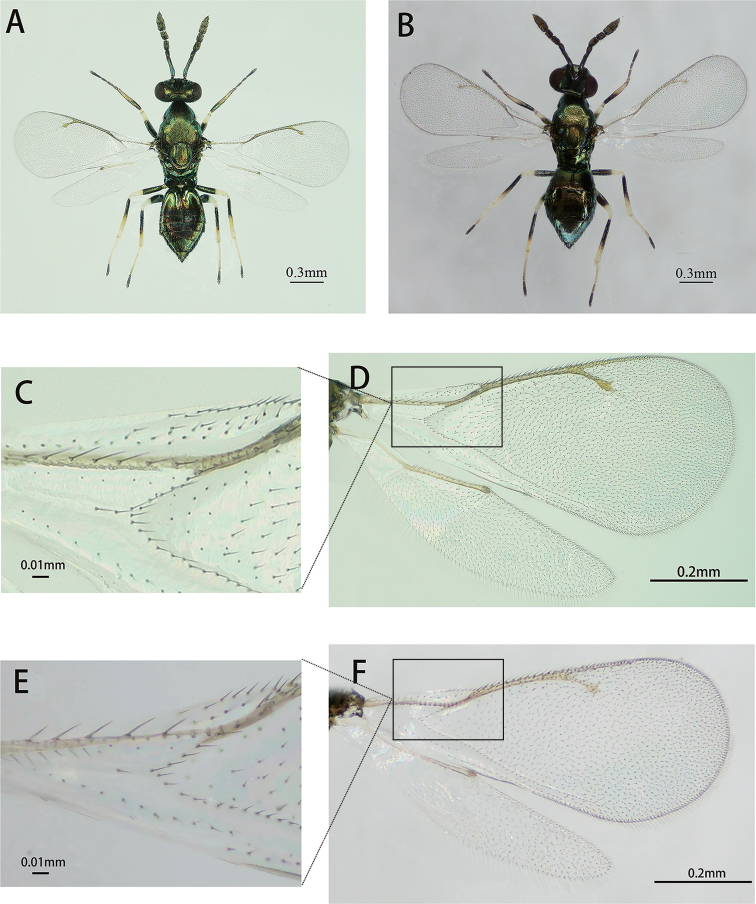
**A** Arrhenotokous female, body, dorsal view **B** Thelytokous female, body, dorsal view **C** Arrhenotokous female, right forewing **D** Arrhenotokous female, right fore and hind-wings **E** Thelytokous female, right forewing **F** Thelytokous female, right fore and hind-wings.

(Fig. 2A). The arrhenotokous female was similar to the thelytokous female in morphological characteristics (Table 2). We only found a little difference on fore- and hind-wings between arrhenotokous and thelytokous *D.wani* (Figs. 2A, B). For the arrhenotokous and thelytokous females, the forewing with denser setae overall, the costal cell with 2 ~ 4 rows and 2 rows of setae on dorsal surface, respectively and basal cell with 15 ~ 21 hairs and 10 ~ 15 hairs, respectively (Figs 2C-2F, indicated by squares). The setation beneath the marginal vein of the hind-wing of the arrhenotokous female (Fig. 2D) is denser than the same area of the thelytokous female (Fig. 2F).

**Table 2.** Comparison of morphological of thelytokous and arrhenotokous females.

Portion	Thelytokous female	Arrhenotokous female
Antenna	Scape 3.3× as long as broad	Scape 3.9× as long as broad
	Pedicel 1.8× as long as broad	Pedicel 2.1× as long as broad
	F1 1.5× as long as broad	F1 1.7× as long as broad
	F2 1.3× as long as broad	F2 1.4× as long as broad
	Clava 2.3× as long as broad	Clava 2.2× as long as broad
	F1 1.1× as long as F2	F1 1.1× as long as F2
	Clava 1.2× as long as scape	Clava 1.2× as long as scape
	and 2.2× as long as F2	and 1.9× as long as F2
Forewing	SMV:MV:PMV:STV =26:42:22:20.	SMV:MV:PMV:STV=44:64:24:21.
Head	POL 2.7× as long as OOL.	POL 2.6× as long as OOL.
Metasoma	Gaster 1.5× as long as broad.	Gaster 1.6× as long as broad.
Ratio of gaster to ovipositor	2.7 ± 0.2	2.6 ± 0.3
Body length	1.0–1.9 mm	0.9–1.8 mm

### Molecular recognition

#### COI gene

There were 23 variable sites with 21 parsimony informative sites of thelytokous strain and seven variable sites with four parsimony informative sites of arrhenotokous strain in 744 bp. Base insertion, deletion and stop codons were not found in all sequences. The identities of the COI gene sequence of arrhenotokous *D.wani* with seven haplotypes were 95 ~ 96% with *D.wani* (MF590062), 90% with *D.pulchripes* (DQ390435), *D.isaea* (DQ149173) and *D.pachyneurus* (DQ149193) and 87% with *D.bimaculatus* (DQ149161) in GenBank.

**Figure 3.**
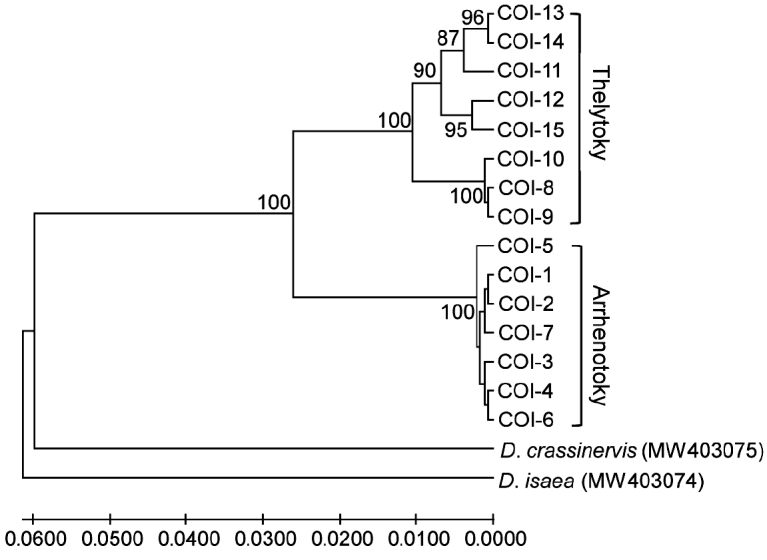
Phylogenetic tree of *Diglyphuswani* and related *Diglyphus* species, based on COI gene of the primers COΙSF/COΙ2613 amplification. COI-1 ~ COI-15 were indicated as the COI gene haplotypes of *D.wani*.

A total of 15 haplotypes (COI-1 ~ COI-15) was found, seven (COI-1 ~ COI-7) of the arrhenotokous strain and eight (COI-8 ~ COI-15) of the thelytokous strain. The haplotype sequences of *D.wani* and *D.isaea* and *D.crassinervis* were uploaded to GenBank (accession numbers: MW403074, MW403090). *Diglyphuswani* individuals showed intraspecific genetic variation (Table 3). The mean sequence divergence was 0.052 between two strains and 0.112 ~ 0.134 between related *Diglyphus* species. Phylogenetic analysis showed *D.wani* species formed two major branches, which were thelytokous and arrhenotokous strains, respectively (Fig. 3).

**Table 3.** The mean genetic divergence between two strains of *D.wani* and related *Diglyphus* species.

Number	Species	COI				ITS1				ITS2			
		1	2	3	4	1	2	3	4	1	2	3	4
1	Arrhenotokous *D.wani*												
2	Thelytokous *D.wani*	0.052				0.010				0.007			
3	*D.crassinervis*	0.128	0.112			0.265	0.265			0.082	0.076		
4	*D.isaea*	0.134	0.113	0.123	—	0.241	0.238	0.265	—	0.072	0.064	0.107	—

#### ITS1 gene

The ITS1 gene sequences of arrhenotokous and thelytokous strains were 617 bp and 636 ~ 680 bp, respectively. A total of eight variation sites were detected in the thelytokous strain and two parsimony informative sites (excluding gaps) were found. The sequences exhibited characters of internal repeat sequences. Then the ITS1 gene sequences of arrhenotokous *D.wani* were identified after BLAST in GenBank. The identities of the ITS1 gene sequences of arrhenotokous *D.wani* were 93.96% with *D.isaea* (AY948091.1), 87.19% with *D.crassinervis* (AY948110.1), 88.93% with *D.begini* (AY948107.1) and 82.56% with *D.bimaculatus* (AY948109.1).

**Figure 4:**
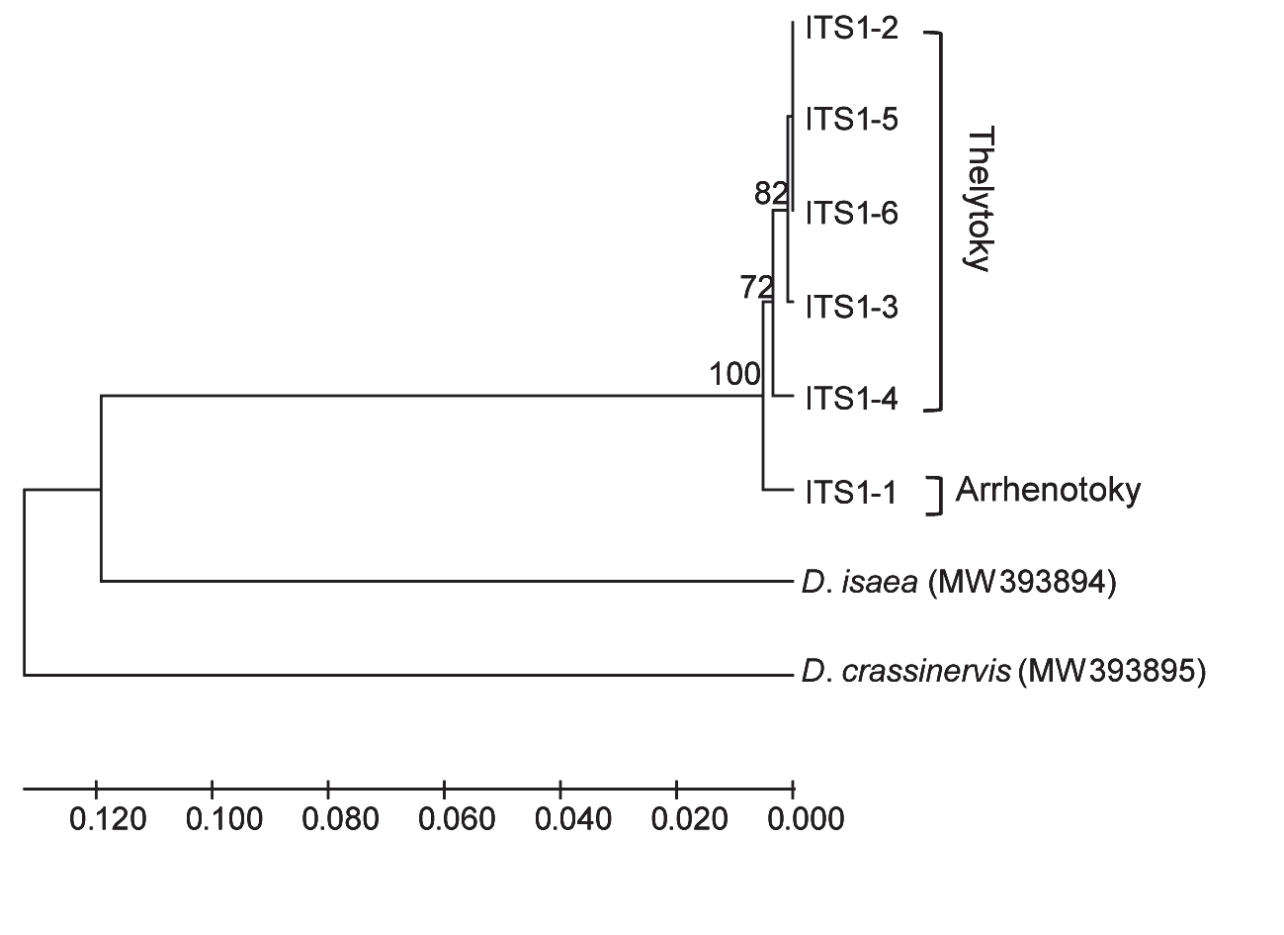
Phylogenetic tree of *Diglyphuswani* and related *Diglyphus* species, based on ITS1 gene of the primers 18sf1/5p8sB1d amplification. ITS1–1 ~ ITS1–6 were indicated as the ITS1 gene haplotypes of *D.wani*.

In comparison with the COI gene, the ITS1 gene showed lower haplotype diversity, showing six haplotypes (ITS1–1 ~ ITS1–6) when gaps were not considered. Of ITS1 gene haplotypes, only one haplotype (ITS1–1) was found in the arrhenotokous strain; however, the thelytokous strain had five haplotypes (ITS1–2 ~ ITS1–6). The haplotype sequence of *D.wani*, *D.isaea* and *D.crassinervis* were uploaded to GenBank (accession number: MW393894, MW393901). The mean sequence divergence was 0.010 between two strains and 0.241 ~ 0.265 between related *Diglyphus* species (Table 3). Similar to the COI analysis, *D.wani* species formed two major branches, which were thelytokous and arrhenotokous strains, respectively, separated from *D.isaea* and *D.crassinervis* (Fig. 4).

#### ITS2 gene

The ITS2 sequence length of arrhenotokous and thelytokous strains was 389 bp and 388 bp, respectively. Sequence analysis showed three variation sites and no parsimony informative sites when analysing sequences of two strains integrally. The identities of the ITS2 sequences of arrhenotokous species were 87% with *D.begini* (MH818358.1) and 77% with *D.isaea* (MH818359.1) in GenBank.

**Figure 5.**
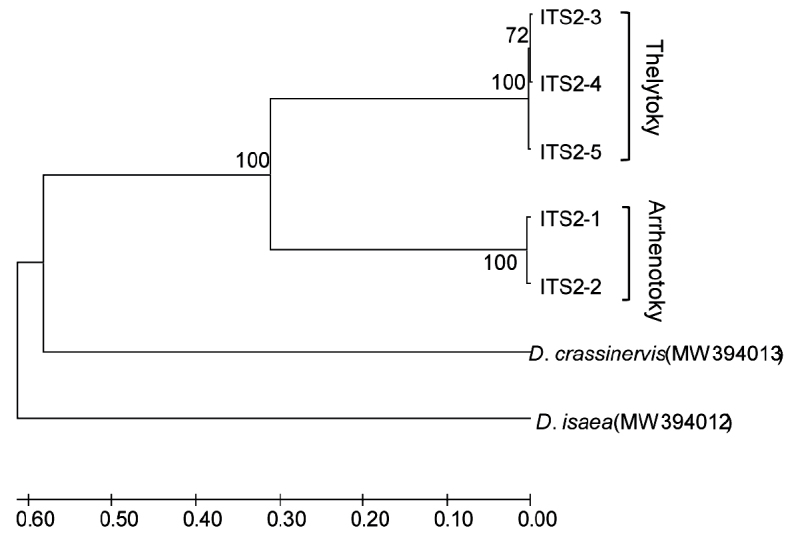
Phylogenetic tree of *Diglyphuswani* and related *Diglyphus* species, based on ITS2 gene of the primers ITS2F/ITS2R amplification. ITS2–1 ~ ITS2–5 were indicated as the ITS2 gene haplotypes of *D.wani*.

A total of five haplotypes (ITS2–1 ~ ITS2–5) was found when gaps were not considered. Amongst them, there were two haplotypes (ITS2–1 ~ ITS2–2) of the arrhenotokous strain and three haplotypes (ITS2–3 ~ ITS2–5) of the thelytokous strain. The haplotype sequence of *D.wani*, *D.isaea* and *D.crassinervis* were uploaded to GenBank (accession numbers: MW394012, MW394018). The mean sequence divergence was 0.007 between two strains and 0.064 ~ 0.107 between interspecies variation (Table 3). The phylogenetic relationship of the ITS2 region is shown in Fig. 5. The two strains of *D.wani* form two branches including arrhenotokous and thelytokous strains, respectively, which grouped with *D.crassinervis*.

#### 28S gene

The length of the 28S sequences from two strains of *D.wani* was 529–530 bp in all individuals and only one site had undergone C and T transition mutually. The identities of arrhenotokous species were 100% with *D.isaea* (MH169044.1), 99% with *D.begini* (MH814438.1) and *D.minoeus* (DQ390423.1) and 98% with *D.pachyneurus* (DQ390424.1) in GenBank.

**Figure 6.**
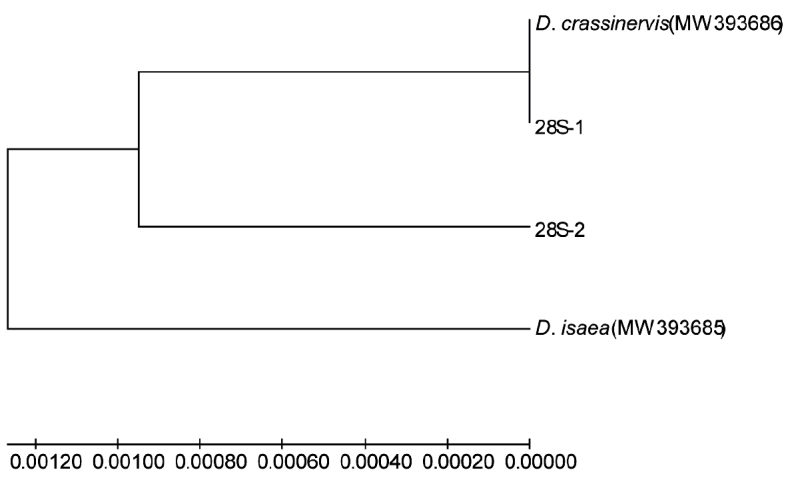
Phylogenetic tree of *Diglyphuswani* and related *Diglyphus* species, based on 28S gene of the primers D2F/D2R amplification. 28S-1 and 28S-2 were indicated as the 28S gene haplotypes of *D.wani*.

Two haplotypes were found within two strains. The haplotypes sequences of *D.wani*, *D.isaea* and *D.crassinervis* were uploaded to GenBank (accession numbers: MW393685, MW393688). Nevertheless, two strains shared a common haplotype. Haplotype 28S-1 was across all arrhenotokous and partial thelytokous individuals and haplotype 28S-2 was included in the other thelytokous individuals. The phylogenetic analysis showed haplotype 28S-1 and *D.crassinervis* formed one branch due to the same sequences, then clustered with 28S-2 and *D.isaea* (Fig. 6).

## Discussion

In many insect orders, both arrhenotokous and thelytokous strains can be commonly found, such as Hemiptera and Psocodea (Bøcher and Nachman 2011; Yang et al. 2015; van der Kooi et al. 2017). In Hymenopteran parasitoids, species with arrhenotoky and thelytoky are not rare (Schneider et al. 2002; Adahi-Hagimori et al. 2011; Gebiola et al. 2012). However, systematic taxonomical studies on different strains of the conspecific parasitoids are relatively few. Our results indicated that *D.wani* confirmed both arrhenotokous and thelytokous reproduction modes existed in this species. Besides, the current study is the first directly targeting the morphological and molecular identification of arrhenotokous and thelytokous strains of *D.wani*.

In general, arrhenotokous and thelytokous strains of Hymenopteran parasitoids are similar in morphology. They may differ in body colour, body length, eyes, wing size and shape, spermathecae and ovaries occasionally (Reineke et al. 2004; Reumer et al. 2013; Petrović et al. 2015; Gebiola et al. 2017). The important distinguishing features we found in the fore- and hind-wings provided an enormous convenience for quickly distinguishing two strains of *D.wani*. These features were mainly on the density of setae in the costal cell and basal cell. At the same time, based on COI gene, ITS1 gene and ITS2 gene, the sequences divergence between *D.wani* and related *Diglyphus* species was far greater than inter-strains divergence. Phylogenetic analysis results showed that the COI gene, ITS1 gene and ITS2 gene can distinguish two strains of *D.wani* according to the cluster of phylogenetic trees. The COI gene was the best maker to distinguish the two strains of *D.wani* due to a greater sequence divergence, followed by the ITS gene and the 28S gene cannot distinguish them, because the sequence conservation of the ITS gene and 28S gene was significantly higher than that of the COI gene. Thus, the COI gene can be used as a more effective marker to judge different strains of *D.wani*, as well as strains of *Tetrastichuscoeruleus* (Hymenoptera: Eulophidae) (Reumer et al. 2013).

Although Hebert et al. (2003) analysed that COI-based sequences divergences amongst the 13320 species and argued 2% gene divergence possessing at least 400 bp of COI sequence was employed as a threshold for species diagnosis, it is controversial (Mallet and Willmott 2003), especially for different strains of a species. Besides, the length of the sequence will affect the delimitation of this threshold (Yang et al. 2017). Yang et al. (2017) reported the COI gene divergence of two strains of *N.formosa* were 2.3% and 3.9% when using a primer combination (COI1 and COI2) to amply the 520 bp region and another primer combination (LCO1490 and HCO2198) to amply the 710 bp region, respectively. The COI gene sequence divergence between two strains of *T.coeruleus* was 3.3% ~ 3.7% according to 991 bp of the sequence (Reumer et al. 2013). In this study, the gene divergence between two strains of *D.wani* was more than 2%, based on the 744 bp of COI sequence. Therefore, the threshold of 2% COI gene divergence is not available for species delimitation in some situations (Murata et al. 2009; Reumer et al. 2013; Yang et al. 2017; Fujie et al. 2019). Furthermore, some species obtaining two strains may have become a genetically-distinct complex or cryptic species on account of a high level of genetic divergence. Cryptic species are at least superficially morphologically indistinguishable, but have distinct genetic structures (Bickford et al. 2007). Based on the COI gene, the sequence divergence between two strains of *N.formosa* from China was 2.3%, amongst which the thelytokous strain had a closer genetic relationship with thelytokous *N.formosa* from Japan (Yang et al. 2017). However, the sequence divergence between thelytokous and arrhenotokous strains of *N.formosa* in Japan is 8.6% (Adachi-Hagimori et al. 2011). Molecular analyses suggested that *N.formosa* could be a complex of at least two cryptic species, the first one including the thelytokous strain from Japan and two strains of *N.formosa* from China, the second one from Japan which was arrhenotoky (Yang et al. 2017, unpublished data).

In general, a crossing experiment was carried out to verify whether there were reproductive barriers between the two strains of a parasitoids (Arakaki et al. 2000; Kraaijeveld et al. 2009; Reumer et al. 2013). Thelytokous *Leptopilinaclavipes* (Hymenoptera: Figitidae) was infected with *Wolbachia* and males were produced by antibiotic treatments (Kraaijeveld et al. 2009). The discoveries were that arrhenotokous males and males derived from thelytokous strains can mate with thelytokous and arrhenotokous females (Kraaijeveld et al. 2009). In contrast, in the parasitoid *T.coeruleus* whose thelytoky is the result of infection with *Wolbachia*, although thelytokous females were attractive to arrhenotokous males, thelytokous females were unreceptive to males (Reumer et al. 2014). For thelytokous *D.wani*, we did not detect thelytoky-inducing endosymbionts reported previously; moreover, high temperature or antibiotic treatment for five generations did not reverse the thelytokous reproductive pattern to produce males (unpublished data). We also conducted laboratory crossing between strictly thelytokous females and arrhenotokous males of *D.wani*; however, no male progeny was produced (unpublished data).

Previous studies demonstrated thelytokous *D.wani* had high fecundity and three types of host-killing behaviour (Ye et al. 2018). The arrhenotokous strains of *D.wani* also exhibited strong biocontrol potential and the two strains of *D.wani* most notably attacked agromyzid leafminers, especially against *C.horticola*, *L.sativae* and *L.huidobrensis* in the field. In the follow-up studies, it is particularly important to compare and evaluate the biological characteristics of the two strains and to clarify control efficiency when releasing one strain alone, releasing two strains together or releasing them with other parasitoids jointly.

## Supplementary Material

XML Treatment for Diglyphus wani
